# Comparative evaluation of the clinical laboratory-based Intermountain risk score with the Charlson and Elixhauser comorbidity indices for mortality prediction

**DOI:** 10.1371/journal.pone.0233495

**Published:** 2020-05-21

**Authors:** Gregory L. Snow, Joseph R. Bledsoe, Allison Butler, Emily L. Wilson, Susan Rea, Sarah Majercik, Jeffrey L. Anderson, Benjamin D. Horne

**Affiliations:** 1 Office of Research, Intermountain Healthcare, Salt Lake City, Utah, United States of America; 2 Emergency Department, Intermountain Medical Center, Salt Lake City, Utah, United States of America; 3 Department of Emergency Medicine, Stanford University School of Medicine, Stanford, California, United States of America; 4 Pulmonary and Critical Care Division, Department of Medicine, Intermountain Medical Center, Salt Lake City, Utah, United States of America; 5 Care Transformation, Intermountain Healthcare, Salt Lake City, Utah, United States of America; 6 Intermountain Medical Center Heart Institute, Salt Lake City, Utah, United States of America; 7 Cardiology Division, Department of Medicine, University of Utah School of Medicine, Salt Lake City, Utah, United States of America; 8 Division of Cardiovascular Medicine, Department of Medicine, Stanford University School of Medicine, Stanford, California, United States of America; Osakidetza Basque Health Service, SPAIN

## Abstract

**Background:**

The Charlson and Elixhauser comorbidity indices are mortality predictors often used in clinical, administrative, and research applications. The Intermountain Mortality Risk Scores (IMRS) are validated mortality predictors that use all factors from the complete blood count and basic metabolic profile. How IMRS, Charlson, and Elixhauser relate to each other is unknown.

**Methods:**

All inpatient admissions except obstetric patients at Intermountain Healthcare’s 21 adult care hospitals from 2010–2014 (N = 197,680) were examined in a observational cohort study. The most recent admission was a patient’s index encounter. Follow-up to 2018 used hospital death records, Utah death certificates, and the Social Security death master file. Three Charlson versions, 8 Elixhauser versions, and 3 IMRS formulations were evaluated in Cox regression and the one of each that was most predictive was used in dual risk score mortality analyses (in-hospital, 30-day, 1-year, and 5-year mortality).

**Results:**

Indices with the strongest mortality associations and selected for dual score study were the age-adjusted Charlson, the van Walraven version of the acute Elixhauser, and the 1-year IMRS. For in-hospital mortality, Charlson (c = 0.719; HR = 4.75, 95% CI = 4.45, 5.07), Elixhauser (c = 0.783; HR = 5.79, CI = 5.41, 6.19), and IMRS (c = 0.821; HR = 17.95, CI = 15.90, 20.26) were significant predictors (p<0.001) in univariate analyses. Dual score analysis of Charlson (HR = 1.79, CI = 1.66, 1.92) with IMRS (HR = 13.10, CI = 11.53, 14.87) and of Elixhauser (HR = 3.00, CI = 2.80, 3.21) with IMRS (HR = 11.42, CI = 10.09, 12.92) found significance for both scores in each model. Results were similar for 30-day, 1-year, and 5-year mortality.

**Conclusions:**

IMRS provided the strongest ability to predict mortality, adding to and attenuating the predictive ability of the Charlson and Elixhauser indices whose mortality associations remained statistically significant. IMRS uses common, standardized, objective laboratory data and should be further evaluated for integration into mortality risk evaluations.

## Introduction

Charlson et. al. [1] and Elixhauser et. al. [2] previously proposed comorbidity measures to predict mortality using diagnoses of chronic diseases and health conditions. These risk scores aim to encapsulate the overall health of a patient based on the patient’s comorbidity history. Both scores rely on International Statistical Classification of Diseases and Related Health Problems (ICD) coding to define the various comorbidities, summarizing conditions the patient has currently or was diagnosed with in the past even if they are in remission. These comorbidity indices provide a useful and practical summary of risk information that is clinically attractive because they summarize overall patient status through a common-sense approach. Further, they use data elements that are readily available to clinicians in the electronic health record (EHR), or at the conclusion of the history and physical exam. The scores show their utility through their common application in summarizing patient health status in research projects and their frequent utilization in administrative arenas, and the widespread consideration of comorbidities in clinical practice.

Originally, the Charlson score was published with a set of weights for each comorbidity [1]. It was based on a training set of 607 general medical patients and was derived for predicting in-hospital and 1-year mortality. The Elixhauser index, in contrast, was simply published as a list of comorbidities with no weights [2]. It was based on 1.7 million patients, and the outcomes of hospital length of stay and in hospital mortality were used. Over the many years since their original derivations [1,2], variants of these comorbidity measures have been proposed and at times used in place of the original parameterizations, including versions adjusting for age or using different weighting schemes.

More recently, the Intermountain Risk Score (IMRS) was proposed by Horne et. Al [3]. IMRS is a laboratory-based non-comorbidity score that uses common standard-of-care clinical laboratory panels (i.e., complete blood count and basic metabolic profile) along with age and sex to predict mortality at 30 days, 1 year, and 5 years [3]. This tool requires the patients to have a blood draw and laboratory analysis, but it does not depend on examining pre-existing accurate medical records, conducting a physical exam, or delving into a patient’s memory of their medical history. The IMRS models were all originally derived based on71,921 hospitalized patients and validated in 47,458 independent patients drawn from the same population. They were also validated in 16,372 NHANES III participants and 2,558 cardiac catheterization patients [3]. Many subsequent validations have been performed [4–8].

To our knowledge, no formal comparison of these three risk scores has been conducted. The aims of this study were to evaluate the predictive abilities for mortality of multiple variants of the Charlson index, the Elixhauser index, and IMRS among inpatients and to examine the association with mortality of a combination of the comorbidity scores and IMRS. The primary objective was to determine which score is a superior mortality predictor and whether the scores are complementary or if one fully accounts for the risk prediction ability of the others.

## Materials and methods

Study patients included all adult patients (age 18 years and over) with inpatient admissions at one of twenty-three Intermountain Healthcare hospitals between 2010 to 2014. Women admitted for labor and delivery were excluded. Patients admitted more than once during the study period were included only once using their most recent admission. This cohort definition ensured that the study period provided a large sample that did not overlap with the training or test cohorts used in deriving and validating IMRS while allowing 5 years of follow-up for the majority of the cohort through the end of 2018. A total of N = 197,680 patients were included in the final study cohort. This study was approved by the Intermountain Healthcare Institutional Review Board as a data-only historical records review study with a waiver of consent.

The Charlson and Elixhauser scores were computed for each patient using multiple previously-derived versions based on the ICD codes in the electronic medical record [1,2,9–13]. Because the study period ended prior to the implementation of ICD-10 coding, only ICD-9 codes were used in the definition of comorbidities. The IMRS sex-specific models were computed based on the first laboratory results (basic metabolic profile and complete blood count panels) after the admission, as well as age. IMRS scores are computed as the sum of scalar weightings for each component variable and depend on the value of each component and the time frame for risk prediction [3]. The weightings are provided in Table 2 of the 2009 derivation and initial validation paper [3], but have also been provided in the S1 Appendix. IMRS can be computed for 30-day, 1-year, or 5-year mortality and the scores are computed using different weightings for females and males [3]. The basic metabolic profile components utilized in IMRS included sodium, potassium, calcium, bicarbonate, glucose, and creatinine. Complete blood count parameters that were used to compute IMRS included hematocrit, white blood cell count (WBC), platelet count, mean corpuscular volume (MPV), red cell distribution width (RDW), mean corpuscular hemoglobin concentration (MCHC), and mean platelet volume (MPV). These laboratory tests were completed for each patient for clinical purposes and the first available during the index hospital admission or, where necessary, within 6 months prior to the admission.

The outcomes of interest were in hospital mortality, 30-day mortality, 1-year mortality, and 5-year mortality utilizing time to death or censoring in survival analysis. Mortality for each time point was assessed using death records from the Intermountain electronic medical record as well as searching the United States Social Security Administration death master file and Utah death certificates to capture deaths of patients that occurred outside of the healthcare system.

Three versions of the Charlson Comorbidity Score were computed for each patient: the original weighted count of comorbidities [1], an age adjusted Charlson Comorbidity score that includes the same weights as the original but adds additional points for age [9], and a simple count of the number of comorbidities from the Charlson list (giving every comorbidity equal weight) [10].

Further, 8 versions of the Elixhauser score were calculated: 4 based on a historical record of comorbidities and the same 4 using only acute comorbidities. In the historical versions, a comorbidity was counted if the patient had a qualifying ICD code recorded any time at or before their hospital admission, while the acute version was limited to the comorbidities present based on ICD codes recorded for the index hospitalization only. The 4 score variants were the raw count of comorbidities [2], the weighting proposed by van Walraven [11], and the 2 proposed by Thompson [12].

IMRS models were derived for predicting 30-day mortality, 1-year mortality, and 5-year mortality and utilize distinct weightings of each component variable depending on which time point is being evaluated [3].

We evaluated the predictive ability of each score by including the score as the only predictor in a Cox regression model with each of the outcomes of interest as the response variable. Each model is summarized by the area under the ROC curve measured by the c-statistic that was calculated in R using a logistic regression-based procedure and its 95% confidence intervals (CI) were computed by bootstrapping. The Charlson score variant with the consistently highest c-statistic for mortality at each of the time points was used for analyses comparing the predictive ability of Charlson to Elixhauser and IMRS models, and the other Charlson scores were excluded. The same selection process was used to choose one Elixhauser model and one IMRS as the best predictive score from that type to use in comparative analyses between the three types of scores.

Tertiles for each score were also computed and used as predictor variables in separate models to represent a scale of Mild/Moderate/Severe risk of mortality instead of the original continuously distributed linear score variable. Tertiles for IMRS variables were computed separately for females and males because IMRS is distributed differently in each sex and were then combined into one tertile variable, which allows IMRS tertiles to be comparable to Charlson and Elixhauser tertiles. Cox regression was used to evaluate the survival of patients in each tertile in models entering two scores or what we can call dual risk score analyses. Models entering IMRS also adjusted for sex in order to remove any residual differences between females and males that categorizing the tertiles separately had not resolved. Models calculating final c-statistics for Charlson, Elixhauser, and IMRS also entered age and sex, regardless of whether a risk score utilized age or sex in the prediction model.

Dual score regression models were evaluated to examine comparisons of two of the three types of scores simultaneously, including a Charlson index score, an Elixhauser score, and an IMRS. The two score models were used to determine whether each risk score provided additional information beyond the other scores, with the following three combinations of two variables evaluated separately: a model with a Charlson score and an Elixhauser score, a model with a Charlson score and an IMRS, and a model with an Elixhauser score and an IMRS.

Comparisons of Cox models entering one score vs. two scores were performed by examining the -2 log likelihood of each model in which the continuous variables of the scores were entered. Similar methods were used to evaluate the three scores in single score and dual score models of continuous valued scores, with HRs calculated per unit increase of each score. Additionally, those dual score models were expanded by adjusting in Cox regression for two scores plus sex, race, length of index hospitalization, and the comorbidities or laboratory variables not included in the two scores entered in the model. That is, when Charlson and Elixhauser were entered, multivariable adjustment included the component laboratory results used to create IMRS, including hematocrit, white blood cell count, platelet count, mean corpuscular volume, mean corpuscular hemoglobin concentration, red cell distribution width, mean platelet volume, sodium, potassium, bicarbonate, calcium, glucose, and creatinine). When Charlson and IMRS were entered in the Cox model, the variables listed in S1 Table (the components of Elixhauser indices) that are not components of Charlson were also entered. When Elixhauser and IMRS were entered, the comorbidity variables listed in Table 1 (the components of Charlson indices) that are not in Elixhauser were also entered. The net reclassification improvement index (NRI) was also computed to calculate the relative improvement in prediction that IMRS provided beyond the Charlson or Elixhauser indices for each mortality time point. R (r-project.org) and SAS (v. 9.3, SAS Institute, Cary, NC) were used for study analyses. Nominal statistical significance was set at p≤0.05 using two-sided tests of hypothesis.

**Table 1:** Baseline characteristics of the study population.

Characteristic	Overall (N = 197,680)	IMRS Tertile 1 (n = 73,886)	IMRS Tertile 2 (n = 68,128)	IMRS Tertile 3 (n = 55,666)	p-value
**Age (years), mean±SD**	59±19	46±16	61±16	74±14	<0.001
**Female, % (N)**	52.3% (103,366)	49.6% (36,666)	55.5% (37,811)	51.9% (28,889)	<0.001
**Charlson index (age-adjusted)**	5±4	3±3	5±3	8±4	<0.001
**Elixhauser index (acute, van Walraven)**	3±6	0±4	2±6	6±7	<0.001
**Diabetes with complications**	6.8% (13,496)	2.1% (1,521)	6.8% (4,604)	13.2% (7,371)	<0.001
**Moderate/severe liver disease**	1.4% (2,746)	0.4% (298)	1.3% (901)	2.8% (1,547)	<0.001
**Metastatic carcinoma**	3.5% (6,892)	1.3% (945)	3.3% (2,268)	6.6% (3,679)	<0.001
**AIDS**	0.1% (247)	0.1% (101)	0.1% (87)	0.1% (59)	0.29
**Chronic pulmonary disease**	36.8% (72,712)	30.0% (22,144)	37% (25,221)	45.5% (25,347)	<0.001
**Connective tissue disease**	6.0% (11,892)	3.0% (2,230)	6.4% (4,327)	9.6% (5,335)	<0.001
**Myocardial infarction**	10.0% (19,712)	4.4% (3,285)	9.5% (6,485)	17.9% (9,942)	<0.001
**Paraplegia and hemiplegia**	3.0% (5,901)	1.9% (1,390)	3.2% (2,148)	4.2% (2,363)	<0.001
**Cerebrovascular disease**	15.3% (30,152)	7.1% (5,245)	15.1% (10,283)	26.3% (14,624)	<0.001
**Heart failure**	16.5% (32,529)	5.0% (3,700)	14.8% (10,060)	33.7% (18,769)	<0.001
**Dementia**	2.4% (4,682)	0.4% (328)	1.8% (1,254)	5.6% (3,100)	<0.001
**Peripheral vascular disease**	13.7%(27,135)	5.3% (3,945)	13.0%(8,840)	25.8%(14,350)	<0.001
**Moderate/severe renal disease**	12.9% (25,520)	3.5% (2,613)	11.3% (7,690)	27.3% (15,217)	<0.001
**Peptic ulcer disease**	7.8% (15,340)	4.7% (3,482)	7.7% (5,258)	11.9% (6,600)	<0.001
**Cancer**	14.5% (28,652)	7.9% (5,871)	14.3% (9,735)	23.4% (13,046)	<0.001
**Mild liver disease**	12.4% (24,544)	9.6% (7,109)	13.6% (9,257)	14.7% (8,178)	<0.001
**Diabetes without complications**	18.0% (35,644)	10.4% (7,705)	20.4% (13,915)	25.2% (14,024)	<0.001

P-values compared the association of age, sex, and the Charlson comorbidities across tertiles of the 1-year IMRS. For Elixhauser characteristics stratified by IMRS, see S1 Table. Note that for Elixhauser, acute refers to diagnoses at the index admission only.

## Results and discussion

Overall, N = 197,680 patients were included as subjects in this study, with 52.3% females and mean age 59 years. Race was predominantly white (91%), with 0.9% of African ancestry, 0.8% Asian, 0.9% Native Hawaiian or other Pacific Islander, and 0.6% American Indian or Alaska Native (3.9% were other or mixed race and 2.0% were unavailable). Ethnicity was 4.7% Hispanic or Latino. Other baseline characteristics are summarized in Table 1, overall and by IMRS. These characteristics show a powerful trend across IMRS tertiles for almost all comorbidity variables (except AIDS), indicating that IMRS potentially captures important variation related to comorbidities although it utilizes complete blood count and basic metabolic profile components with no comorbidity diagnoses. S1 Table provides similar information by IMRS tertiles for Elixhauser characteristics. S2 Table presents the score thresholds of each Charlson, Elixhauser, and IMRS tertile and Table 2 provides the percentage of patients in each tertile and the percentage in the tertile who deceased.

**Table 2:** Distribution of subjects and mortality by score values for each model.

Score, Model	1^st^ Tertile: subjects; mortality	2^nd^ Tertile: subjects; mortality	3^rd^ Tertile: subjects; mortality	C-statistic^b^
***Charlson index*** ^a^				
**Original**	52%; 9.7%	23%; 21.4%	25%; 46.8%	0.75
	103,162; 10,043	44,792; 9,605	49,726; 23,264	
**Age adjusted**	44%; 5.5%	28%; 18.8%	29%; 49.1%	0.81
	86,246; 4,774	54,880; 10,343	56,554; 27,795	
**Raw sum**	56%; 10.3%	15%; 22.0%	29%; 43.7%	0.74
	110,781; 11,451	30,040; 6,605	56,859; 24,856	
***Elixhauser index*** ^a^				
**Historic, raw sum**	38%; 9.7%	35%; 18.9%	27%; 42.5%	0.73
	75,574; 7,293	69,001; 13,054	53,105; 22,565	
**Historic, van Walraven**	36%; 8.8%	33%; 15.5%	31%; 43.3%	0.75
	71,441; 6,285	64,788; 10,034	61,451; 26,593	
**Historic, Thompson 29**	42%; 9.4%	25%; 15.0%	33%; 42.4%	0.75
	83,168; 7,821	49,212; 7,401	65,300; 27,690	
**Historic, Thompson 30**	38%; 9.1%	30%; 14.9%	33%; 42.3%	0.75
	74,411; 6,737	58,318; 8,688	64,951; 27,487	
**Acute, raw sum**	39%; 8.5%	37%; 20.9%	24%; 44.8%	0.74
	77,255; 6,548	73,582; 15,380	46,843; 20,984	
**Acute, van Walraven**	55%; 9.8%	14%; 22.0%	32%; 42.1%	0.75
	107,877; 10,546	27,150; 5,969	62,653; 26,397	
**Acute, Thompson 29**	36%; 11.4%	31%; 12.6%	33%; 41.8%	0.74
	71,368; 8,155	61,783; 7,789	64,529; 26,968	
**Acute, Thompson 30**	34%; 11.5%	35%; 13.3%	31%; 42.0%	0.74
	66,676; 7,661	68,998; 9,188	62,006; 26,063	
***Intermountain Mortality Risk Score*** ^a^				
**30 day**	35%; 5.9%	34%; 17.3%	32%; 43.8%	0.78
	68,624; 4,042	66,613; 11,497	62,443; 27,373	
**1 year**	37%; 4.8%	34%; 17.1%	28%; 49.9%	0.82
	73,886; 3,529	68,128; 11,628	55,666; 27,755	
**5 year**	35%; 4.3%	34%; 15.7%	30%; 48.7%	0.81
	69,657; 3,013	68,044; 10,691	59,979; 29,208	

^a^Data represent the percentage (first row) and number (second row) of study subjects and the raw mortality in each tertile during the full follow-up time;

^b^C-statistics provided here evaluated the continuous valued risk scores for discrimination of 5-year mortality.

For Elixhauser scores, acute refers to diagnoses at the index admission only, while historic refers to index and previous diagnoses. Note that some tertiles contain substantially more than one third of patients because some scores were skewed toward zero and had many individuals with very low values that were difficult to partition into groups of similar sample size.

In-hospital mortality was recorded in 3.6% of subjects, with 6.9% mortality at 30 days, 12.9% at 1 year, and 20.9% at 5 years. S1 Fig shows the comparison of the areas under the ROC curves (C-statistics) for each of the scores on each of the four mortality outcomes. The areas range from 0.65 (historic Elixhauser raw sum tertiles predicting in hospital mortality) to 0.83 (IMRS 1-year predicting 30-day mortality). Each group of scores has at least one member above 0.7 for each outcome, with many of the scores achieving an area greater than 0.75. In each of the predictors, the linear or continuously-valued score achieved a larger area under the curve than the tertile equivalent.

The age-adjusted Charlson score had a higher c-statistic for all outcomes (c = 0.719, 0.774, 0.801, 0.811 for in-hospital, 30-day, 1-year, and 5-year mortality, respectively) than the originally published (c = 0.681, 0.727, 0.750, 0.749) and raw count versions (c = 0.675, 0.717, 0.739, 0.742), with the original score doing as well or better than the raw count. The acute Elixhauser scores tended to outperform the historic Elixhauser scores, with the difference diminishing as time to the outcome increased. The acute Elixhauser performed similarly to the historic Elixhauser for the 5-year mortality outcome. The raw count of Elixhauser comorbidities consistently had a lower area (historic: c = 0.661, 0.701, 0.722, 0.726 for in-hospital, 30-day, 1-year, and 5-year mortality, respectively; acute: 0.754, 0.759, 0.753, 0.740) than the 3 weighted versions, but there was no clear preferred weighting among the other 3 (S1 Fig). Because the acute van Walraven version (c = 0.783, 0.789, 0.776, 0.748) had higher c-statistics in three of the four mortality endpoints (historic: c = 0.696, 0.739, 0.758, 0.754) and the tertiles of van Walraven had higher c-statistics in all four mortality time points (S1 Fig), this model was chosen to represent the Elixhauser models. The 3 versions of the Intermountain Risk score outperform the other comorbidity scores in essentially every category (the exceptions being the linear 5-year IMRS as a predictor of in-hospital mortality vs. linear Elixhauser, and both linear and tertile 30-day IMRS vs. age-adjusted Charlson for 1-year and 5-year mortality). The 1-year IMRS model (c = 0.821, 0.832, 0.827, 0.817 for in-hospital, 30-day, 1-year, and 5-year mortality, respectively) did the best for 30-day, 1-year, and 5-year outcomes and the 30-day IMRS (c = 0.825, 0.818, 0.800, 0.784) had the highest c-statistic for in-hospital mortality (5-year IMRS: c = 0.775, 0.800, 0.806, 0.805). The age- and sex-adjusted c-statistics and 95% CI for the selected Charlson (age-adjusted), Elixhauser (acute van Walraven), and IMRS (1-year mortality) models are provided in Table 3.

**Table 3:** C-statistics for the selected study risk scores (these results were all age- and sex-adjusted).

	C-Statistic (95% Confidence Interval)			
Risk Score	In-hospital mortality	30-day mortality	1-year mortality	5-year mortality
Continuous Variables				
Charlson	0.719 (0.713, 0.724)	0.774 (0.770, 0.778)	0.801 (0.798, 0.804)	0.811 (0.808, 0.813)
Elixhauser	0.783 (0.778, 0.789)	0.789 (0.785, 0.793)	0.776 (0.773, 0.779)	0.748 (0.745, 0.751)
IMRS	0.821 (0.817, 0.825)	0.832 0.829, 0.835)	0.827 (0.825, 0.830)	0.817 (0.814, 0.819)
Tertile Categories				
Charlson	0.695 (0.690, 0.701)	0.743 (0.739, 0.746)	0.767 (0.765, 0.770)	0.779 (0.776, 0.781)
Elixhauser	0.743 (0.738, 0.747)	0.748 (0.744, 0.751)	0.741 (0.738, 0.744)	0.722 (0.719, 0.724)
IMRS	0.778 (0.774, 0.782)	0.790 (0.787, 0.793)	0.789 (0.786, 0.791)	0.781 (0.779, 0.783)

Due to the tight confidence intervals, all p-values comparing Charlson, Elixhauser, and IMRS c-statistics at each timepoint were p<0.05 for results of the risk scores' continuous values or for their tertiles. The exception was the comparison of the c-statistics for the tertiles of Charlson versus tertiles of IMRS at 5 years. The Charlson index model used here was the age-adjusted version. The Elixhauser model used here was the acute van Walraven version, with acute referring to diagnoses at the index admission only. The IMRS model used here was the 1-year mortality version

The hazard ratios (HRs) with 95% CI for dual score models in Cox regression are provided in Table 4 for all four mortality time points. For in-hospital mortality comparing tertile 3 vs. 1 and 2 vs. 1 in univariable analysis, Charlson had HR = 4.75 and HR = 2.17, Elixhauser had HR = 5.79 and 2.56, and IMRS had HR = 17.95 and 4.94, respectively. These were all reduced when dual score Cox models were constructed, with Charlson+Elixhauser finding tertile 3 vs. 1 and 2 vs. 1 having HR = 2.91 and HR = 1.71 for Charlson and HR = 4.03 and HR = 2.07 for Elixhauser, respectively. For Charlson+IMRS, tertile 3 vs. 1 and 2 vs. 1 had HR = 1.79 and HR = 1.17 for Charlson and HR = 13.10 and HR = 4.24 for IMRS, respectively. For Cox modeling of Elixhauser+IMRS, the tertile 3 vs. 1 and 2 vs. 1 comparisons had HR = 3.00 and HR = 1.73 for Elixhauser and HR = 11.42 and HR = 3.90 for IMRS, respectively. All p-values were p<0.001 in all of these analyses.

**Table 4:** Associations of each score with mortality.

Mortality Timepoint/Cox Regression Model	Charlson HR (95% CI)	Elixhauser HR (95% CI)	IMRS HR (95% CI)
***In-hospital mortality***			
**Univariable**	4.75 (4.45, 5.07)	5.79 (5.41, 6.19)	17.95 (15.90, 20.26)
**Dual score model 1**	2.91 (2.72, 3.12)	4.03 (3.76, 4.33)	----
**Dual score model 2**	1.79 (1.66, 1.92)	----	13.10 (11.53, 14.87)
**Dual score model 3**	----	3.00 (2.80, 3.21)	11.42 (10.09, 12.92)
***30-day mortality***			
**Univariable**	20.76 (18.69, 23.06)	8.61 (8.04, 9.21)	30.40 (26.70, 34.61)
**Dual score model 1**	12.11 (10.87, 13.48)	4.32 (4.03, 4.64)	----
**Dual score model 2**	7.01 (6.27, 7.84)	----	11.03 (9.62, 12.65)
**Dual score model 3**	----	4.07 (3.80, 4.37)	17.22 (15.09, 19.66)
***1-year mortality***			
**Univariable**	14.29 (13.40, 15.24)	6.33 (6.05, 6.62)	15.97 (14.89, 17.14)
**Dual score model 1**	9.25 (8.66, 9.89)	3.43 (3.27, 3.59)	----
**Dual score model 2**	5.98 (5.57, 6.41)	----	6.32 (5.85, 6.82)
**Dual score model 3**	----	3.40 (3.24, 3.56)	9.99 (9.29, 10.74)
***5-year mortality***			
**Univariable**	11.18 (10.83, 11.54)	5.83 (5.69, 5.97)	14.85 (14.32, 15.40)
**Dual score model 1**	7.32 (7.09, 7.56)	3.38 (3.30, 3.46)	----
**Dual score model 2**	4.65 (4.49, 4.81)	----	6.75 (6.48, 7.02)
**Dual score model 3**	----	3.18 (3.11, 3.26)	9.56 (9.21, 9.92)

All hazard ratio (HR) and 95% confidence interval (CI) data are for the comparison of the third tertile of the indicated score compared to the first tertile. All p-values for all scores in all models were p<0.001. The Charlson index model used here was the age-adjusted version. The Elixhauser model used here was the acute van Walraven version, with acute referring to diagnoses at the index admission only. The IMRS model used here was the 1-year mortality version.

Furthermore, examining the ability of IMRS to add additional information beyond the other risk scores, the 1-year IMRS was examined within each tertile of the other scores (S3 Table). For in-hospital mortality, IMRS tertile 3 vs. 1 had HR = 16.19 (95% CI = 13.49, 19.44), HR = 11.56 (CI = 9.08, 14.73), HR = 8.90 (CI = 6.76, 11.70) in Charlson tertiles 1, 2, 3, respectively (all p<0.001). IMRS tertile 3 vs. 1 within tertiles 1, 2, and 3 of the acute Elixhauser (van Walraven) model for in-hospital mortality also had, respectively, HR = 21.05 (CI = 16.86, 26.29), HR = 9.79 (7.27, 13.19), and HR = 8.33 (CI = 7.05, 9.86), with all having p<0.001. Other results for tertile 2 vs. 1 and for 30-day, 1-year, and 5-year mortality are shown in S3 Table.

When the risk prediction tools were examined as continuous variables, results were similar (S4 Table). For example, for in-hospital mortality the Charlson index (HR = 1.15 per +1 score, CI = 1.14, 1.15), Elixhauser (HR = 1.09 per +1 score, CI = 1.08, 1.09), and IMRS (HR = 1.26 per +1 score, CI = 1.25, 1.26) all predicted mortality (p<0.001). In dual score models, Charlson was reduced by Elixhauser (Charlson HR = 1.09 per +1 score, CI = 1.08, 1.10; Elixhauser HR = 1.07 per +1 score, CI = 1.06, 1.07) and IMRS (Charlson HR = 1.04 per +1 score, CI = 1.04, 1.05; IMRS HR = 1.23 per +1 score, CI = 1.22, 1.24), and Elixhauser reduced by IMRS (Elixhauser HR = 1.05 per +1 score, CI = 1.04, 1.05; IMRS HR = 1.22 per +1 score, CI = 1.21, 1.22). Table 5 provides the changes in the -2 log likelihood of dual score models. Further, adjustment for age, sex, length of index hospital stay, and the unique characteristics of the third score (i.e., the score that was not included in each analysis) refined the associations in models entering two scores (S4 Table). The NRI for IMRS compared to Charlson was 0.838 (CI = 0.820, 0.856), 0.834 (CI = 0.820, 0.848), 0.359 (CI = 0.346, 0.372), and 0.301 (CI = 0.290, 0.312) for in-hospital, 30-day, 1-year, and 5-year mortality, and all had p<0.001. Similarly, the NRI for IMRS compared to Elixhauser was 0.225 (CI = 0.202, 0.249), 0.712 (CI = 0.698, 0.726), 0.697 (CI = 0.686, 0.709), and 0.669 (CI = 0.660, 0.679) for those respective time points.

**Table 5:** Cox regression model improvement by IMRS relative to other scores.

Outcome, Cox model	Score entered first into Cox model	-2 log likelihood of single score model	Second score entered	-2 log likelihood of two-score model	Difference^a^
***In-hospital mortality***					
**Model A**^b^	Charlson	-77,640	IMRS	-75,413	-2,227
**Model B**^b^	IMRS	-75,567	Charlson	-75,413	-154
**Model C**^b^	Elixhauser	-77,219	IMRS	-74,853	-2,366
**Model D**^b^	IMRS	-75,567	Elixhauser	-74,853	-714
***30-day mortality***					
**Model A**	Charlson	-159,641	IMRS	-154,676	-4,965
**Model B**	IMRS	-155,705	Charlson	-154,676	-1,029
**Model C**	Elixhauser	-158,266	IMRS	-152,888	-5,378
**Model D**	IMRS	-155,705	Elixhauser	-152,888	-2,817
***1-year mortality***					
**Model A**	Charlson	-295,009	IMRS	-287,584	-7,425
**Model B**	IMRS	-290,647	Charlson	-287,584	-3,063
**Model C**	Elixhauser	-295,306	IMRS	-285,968	-9,339
**Model D**	IMRS	-290,647	Elixhauser	-285,968	-4,679
***5-year mortality***					
**Model A**	Charlson	-475,201	IMRS	-465,313	-9,888
**Model B**	IMRS	-470,792	Charlson	-465,313	-5,480
**Model C**	Elixhauser	-479,046	IMRS	-465,168	-13,878
**Model D**	IMRS	-470,792	Elixhauser	-465,168	-5,624

^a^Differences in -2 log likelihood between the single score and dual score Cox regression models were all highly significant statistically due to the sample size, with p<0.001, thus the magnitude of difference is the more informative result;

^**b**^Model A entered the Charlson index first and then added IMRS to the model and model C did similarly for Elixhauser with IMRS added second, while models B and D added IMRS to the model and then added Charlson or Elixhauser, respectively.

The Charlson index model used here was the age-adjusted version. The Elixhauser model used here was the acute van Walraven version, with acute referring to diagnoses at the index admission only. The IMRS model used here was the 1-year mortality version.

### Summary of findings

Among a very large hospitalized patient population, IMRS added predictive ability for mortality that was not contained in the comorbidity-based Charlson or Elixhauser risk scores. IMRS was individually more predictive of risk than those scores and also was more strongly associated with mortality in regression models that evaluated both IMRS and one of the other two scores. Further, IMRS provided substantial risk information in each tertile of both Charlson and Elixhauser. This was consistently the case for in-hospital, 30-day, 1-year, and 5-year mortality.

### Context of common risk stratification approaches

The Charlson comorbidity index [1,9,10] and Elixhauser comorbidity scores [2,11–13] are commonly utilized methods of summarizing patient health severity and risk of future adverse outcomes. Despite having been developed initially in the 1980’s or tracing their roots to that time, these have stood the test of time and are still relevant today for predicting mortality at different time points in cohorts of hospitalized patients. This is understandable because each organ system contributes meaningfully to the overall health of each individual and chronic diseases or acute events that limit or damage them lead to degradation of health overall. The comorbidity components of the scores are also commonly evaluated in medicine as part of the history and physical examination, and even more today they are available in legacy records through the EHR. As a consequence, these scores are frequently used in administrative and research applications to evaluate patient health status or prognosis or to adjust analyses to determine the individual contribution of a new risk predictor in the context of the Charlson or Elixhauser risk information.

Limitations of these scores, though, include that assessment of comorbidities requires human evaluation of a patient and the reliability and accuracy of a diagnosis of its presence or absence. In most cases, the validity of the diagnoses is not systematically verified. The availability of ICD codes necessary to calculate the Charlson or Elixhauser scores on admission to the hospital remains a limitation of their use in clinical care. Often patients do not have prior records available in the acute setting, creating a challenge for clinicians. Comorbidity scores are not routinely used for the acute care of patients and are often relegated to research purposes. Further, once a comorbidity is diagnosed, that diagnosis does not disappear or become rescinded for most of the comorbidity parameters; thus, over time both Charlson and Elixhauser scores can only increase. This is the case even if medical treatments, surgery or other interventions, or lifestyle changes modify a patient’s prognosis.

### Intermountain mortality risk score

In contrast, IMRS was developed based on a practical parsimonious modeling concept in which commonly ordered, broadly-applicable electronic, standardized, quantitative, objective data elements are used to compose the score [3–7]. IMRS is derived from laboratory results commonly obtained as part of routine care in the emergency department and in other acute and some non-acute settings. It is therefore possible to calculate the IMRS upon ED admission, making it potentially useful for decision making in the acute setting as well as in non-acute and non-urgent clinical processes [14,15]. Not only can the laboratory parameters be re-measured at meaningful time points during a patient’s evaluation and follow-up, but they are also dynamic and can both respond to declines in health as well as reveal improvements in health due to lifestyle changes and medical treatments [8].

As opposed to the comorbidity-based risk scores, IMRS provides a measure of the baseline state of health that may more accurately reflect the activity of underlying diseases. Whereas having a diagnosis of cancer for example may earn a patient points on the Charlson co-morbidity index, it does not accurately reflect the severity of illness or the impact on health that cancer or its treatment portrays. IMRS likely is a more accurate representation of the current effects of disease on overall health, which is plausibly why it more accurately predicts mortality. IMRS components are also inexpensive, even for the limited number of patients who do not have a standard of care indication for the laboratory testing. Further, the complete blood count and metabolic profile laboratory tests from which it is derived have been utilized in medicine for over half a century because they measure the status of physiologically important and meaningful system-wide circulatory and metabolic traits.

IMRS outperformed the other mortality risk scores at each of the measured time intervals in both the linear and tertile risk score models. Given its readily available components, IMRS can be fully automated and operationalized within the EHR. This allows it to be available upon initial laboratory testing, and to be utilized without human calculation in electronic clinical decision support to guide clinician attention to patients with greater health needs [14,15].

This study suggests that evaluation of patient health can be improved by utilizing one of the comorbidity scores along with IMRS, whether for clinical (as possible given the availability of comorbidity information), administrative, or research purposes (e.g., designing and/or analyzing medical studies). As individual measures of future risk, IMRS consistently had the highest statistical effect sizes, although none of the scores fully dominated. Further, in modeling of two scores together the combination of IMRS with one of the comorbidity scores provided the best predictive ability, so choice of which score to use should be based on practical considerations such as which measures of comorbidities are available and how complete or accurate the relevant records are. In part, this includes whether historical or only current assessments are available because acute (i.e., current) comorbidity measures fared better than historical ones for the short-term outcomes, and the weighted and age-adjusted values did better than raw counts of comorbidities.

Interestingly, the 1-year derivation of IMRS had better c-statistics than the 30-day or 5-year IMRS for 30-day and 5-year mortality, respectively. These scores were derived as predictors of mortality at their respective timepoints in a previous Intermountain population [3] and it is a unique finding that the weightings in the 1-year IMRS generalize to other timepoints better than other IMRS derivations. Why this may be the case is uncertain, but may relate to the mortality rate and sample size in the original derivation where the 1-year mortality was 5.4% and 63,190 patients had 1-year outcome data, which mortality was substantially greater than the 1.4% at 30 days but the sample size was of similar magnitude (71,921 patients had 30-day outcome data) [3]. The sample size at 5 years, though, was much lower at 14,214 patients although the mortality was 50.3% [3]. Further investigation is needed to evaluate whether even further optimization can be made to the IMRS components’ weightings for different timepoints.

### Limitations

This was a retrospective observational study, subject to sampling bias and potentially incomplete adjustment for confounding factors based on whether those parameters were measured and how reliable those measurements were. Regression analyses did, however, adjust for a broad range of factors utilized in IMRS and the comorbidity scores. In addition, IMRS was derived on data from the same hospital system in which this study was performed, albeit on different data sets, but the external validity of these specific analyses is unknown (although IMRS itself has been repeatedly externally validated [4,5]). The potential issues may include that the racial and ethnic composition of the study population was homogeneous and that the smoking rate, a common behavioral risk factor in the majority of locales, is the lowest in the US among people in the Utah region. The comorbidities and Charlson and Elixhauser scores were determined from ICD codes in administrative databases, which have been historically noted for their inaccuracies and therefore limit the validity of the dataset in general.

## Conclusions

IMRS, Charlson, and Elixhauser scores predicted in-hospital, 30-day, 1-year, and 5-year mortality. IMRS had the strongest predictive ability and accounted for an important degree of risk information contained in the other scores. The Charlson and Elixhauser scores, although attenuated in their predictive ability by IMRS, did remain significant predictors of mortality in dual score modeling and also were shown to capture some of the risk information contained in IMRS. The superior predictive ability of IMRS suggests that, as a simple predictor of general health, this laboratory-based risk model should be considered for clinical, research, and administrative purposes. Further, for risk evaluations that use a comorbidity score, such as research and administrative applications, IMRS can often be calculated in those situations and could be utilized in conjunction with either a Charlson or Elixhauser model. Further investigation of the use of IMRS, including prospective external validation of use along with a comorbidity score, is required prior to routine clinical use and other applications in medicine.

## Supporting information

S1 Fig C-statistics for each of the Charlson index, Elixhauser score, and IMRS prediction models evaluated in the study. Analysis using tertiles is represented with a triangle and analysis of the linear risk score is represented by a circle. Models were univariable for Charlson and Elixhauser, and for IMRS were adjusted for sex.
(TIF)

S1 Table Elixhauser characteristics overall and by IMRS tertiles. (XLSX)

S2 Table Distribution of the score values for each risk score. (XLSX)

S3 Table Association of IMRS with mortality in each tertile of the Charlson and Elixhauser indices. (XLSX)

S4 Table Association with mortality at each timepoint of continuous values of each risk score in univariable and multivariable analyses. (XLSX)

S1 Appendix Sex-specific values* are used to calculate the Intermountain risk score as the sum of an individual’s corresponding values from each component at a given time point. (DOC)

S1 Checklist STROBE statement—Checklist of items that should be included in reports of *cohort studies*. (DOCX)
