# Supplementary figures and images for: Comparative evaluation of the clinical laboratory-based Intermountain risk score with the Charlson and Elixhauser comorbidity indices for mortality prediction
Source: PLoS One. 2020 May 21;15(5):e0233495. doi: 10.1371/journal.pone.0233495 (PMC7241706; doi:10.1371/journal.pone.0233495)

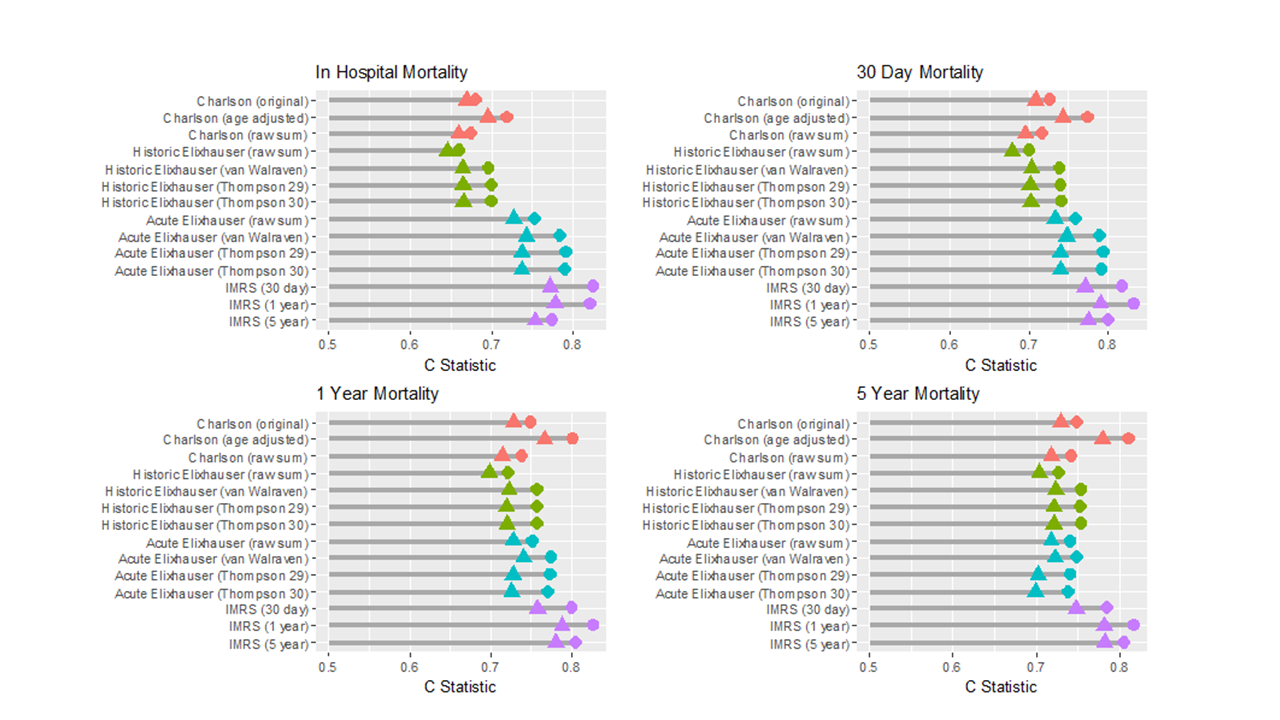

Supplement: S1 Fig — Analysis using tertiles is represented with a triangle and analysis of the linear risk score is represented by a circle. Models were univariable for Charlson and Elixhauser, and for IMRS were adjusted for sex. (TIF) [file pone.0233495.s001.tif]
