# Supplementary material for: Comparative evaluation of the clinical laboratory-based Intermountain risk score with the Charlson and Elixhauser comorbidity indices for mortality prediction
Source: PLoS One. 2020 May 21;15(5):e0233495. doi: 10.1371/journal.pone.0233495 (PMC7241706; doi:10.1371/journal.pone.0233495)
Supplement: S1 Appendix — (DOC) [file pone.0233495.s006.doc]

S1 APPENDIX:

Reprinted from the American Journal of Medicine, 2009/122, Benjamin D. Horne, Heidi T. May, Joseph B. Muhlestein, Brianna S. Ronnow, Donald L. Lappé, Dale G. Renlund, Abdallah G. Kfoury, John F. Carlquist, Patrick W. Fisher, Robert R. Pearson, Tami L. Bair, Jeffrey L. Anderson, Exceptional Mortality Prediction by Risk Scores from

Common Laboratory Tests, Pages 550-558, Copyright (2009), with permission from Elsevier.

<http://www.sciencedirect.com/science/journal/00029343>

doi:10.1016/j.amjmed.2008.10.043

**Table 2**. Sex-specific values* are used to calculate the Intermountain Risk Score as the sum of an individual’s corresponding values from each component at a given time point.

Female s Males

### Component 30-day 1-year 5-year 30-day 1-year 5-year

Hematocrit≤34.6 1 1 2 2 3 3

34.7-38.2 0 0 1 2 2 3

38.3-41.0 0 0 0 1 1 2

41.1-44.1 0 0 0 0 1 1

≥44.2 0 0 1 0 0 0

White Blood Cell Count

≤5.9 0 0 0 0 1 0

6.0-7.3 0 0 0 0 0 0

7.4-8.9 1 0 0 0 1 1

9.0-11.2 2 1 1 2 2 1

≥11.3 4 3 2 4 3 2

Platelet Count≤183 2 1 2 2 1 1

184-220 1 0 0 1 0 0

221-254 1 0 1 0 0 0

255-300 0 0 1 1 1 0

≥301 0 0 1 1 1 1

Mean Corpuscular Volume

≤86.3 0 0 0 0 0 0

86.4-89.1 0 0 0 0 0 0

89.2-91.4 1 0 0 0 0 0

91.5-94.0 0 0 1 0 0 0

≥94.1 1 1 1 1 1 1

Mean Corpuscular Hemoglobin Concentration

≤33.3 1 1 0 1 1 0

33.4-33.8 0 0 0 0 1 0

33.9-34.2 1 0 0 0 0 0

34.3-34.6 0 0 0 0 0 1

≥34.7 0 0 0 0 0 1

Red Cell Distribution Width

≤12.5 0 0 0 0 0 0

12.6-13.0 2 1 1 1 0 0

13.1-13.5 1 1 2 1 1 2

13.6-14.3 3 2 2 2 2 3

≥14.4 4 4 5 3 3 4

Mean Platelet Volume

≤7.5 1 1 1 1 1 0

7.6-8.0 1 0 1 1 0 0

8.1-8.4 1 0 0 2 0 0

8.5-9.1 0 0 0 0 0 0

≥9.2 0 0 0 1 0 0

Sodium≤138 1 1 2 1 1 2

139 0 0 1 1 0 0

140-141 0 0 1 0 0 0

142 0 0 0 1 0 0

≥143 1 1 0 2 1 0

Potassium≤3.7 1 1 1 2 0 0

3.8-3.9 0 0 0 1 0 0

4.0-4.1 0 0 1 1 0 0

4.2-4.4 0 0 0 0 0 0

≥4.5 1 0 1 1 0 0

Bicarbonate≤23 3 1 1 4 2 1

24-25 1 0 0 2 0 0

26 1 0 0 1 0 0

27-28 0 0 0 0 0 0

≥29 2 1 1 1 1 1

Calcium≤8.5 4 3 3 1 2 2

8.6-8.9 2 2 2 0 1 2

9.0-9.2 2 1 1 0 0 1

9.3-9.5 0 0 0 0 1 0

≥9.6 1 1 0 0 0 0

Glucose≤85 1 0 0 1 1 0

86-94 0 0 0 0 0 0

95-104 1 0 1 1 1 0

105-125 1 1 1 2 1 1

≥126 3 2 2 3 2 1

Creatinine≤70.7 0 1 1 2 3 2

70.8-79.6 0 0 1 1 1 1

79.7-88.4 0 0 0 0 1 0

88.5-106.1 1 1 1 0 0 0

≥106.2 2 2 3 2 2 1

Age (years)

18-29 -3 -5 -5 1 0 0

30-39 -2 -1 -1 1 -1 0

40-49 0 0 0 0 0 0

50-59 1 1 1 1 1 1

60-69 2 2 3 1 1 2

70-79 2 3 4 2 2 3

≥80 5 6 8 4 5 7

Sex Female 0 0 0 ----- ----- -----

Male ----- ----- ----- 0 0 0

*Risk models and component values are Copyright © 2006-2008, IHC Health Services, Inc. (freely available for academic use).
